# Systemic Immune Activation Leads to Neuroinflammation and Sickness Behavior in Mice

**DOI:** 10.1155/2013/271359

**Published:** 2013-07-10

**Authors:** Steven Biesmans, Theo F. Meert, Jan A. Bouwknecht, Paul D. Acton, Nima Davoodi, Patrick De Haes, Jacobine Kuijlaars, Xavier Langlois, Liam J. R. Matthews, Luc Ver Donck, Niels Hellings, Rony Nuydens

**Affiliations:** ^1^BIOMED, Hasselt University, Agoralaan C Building, 3590 Diepenbeek, Belgium; ^2^Neuroscience, Janssen Research & Development, A Division of Janssen Pharmaceutica NV, Turnhoutseweg 30, 2340 Beerse, Belgium; ^3^Molecular Imaging, Janssen Research & Development LLC, Welsh & McKean Roads, Spring House, PA 19477-0779, USA

## Abstract

Substantial evidence indicates an association between clinical depression and altered immune function. Systemic administration of bacterial lipopolysaccharide (LPS) is commonly used to study inflammation-associated behavioral changes in rodents. In these experiments, we tested the hypothesis that peripheral immune activation leads to neuroinflammation and depressive-like behavior in mice. We report that systemic administration of LPS induced astrocyte activation in transgenic GFAP-luc mice and increased immunoreactivity against the microglial marker ionized calcium-binding adapter molecule 1 in the dentate gyrus of wild-type mice. Furthermore, LPS treatment caused a strong but transient increase in cytokine levels in the serum and brain. In addition to studying LPS-induced neuroinflammation, we tested whether sickness could be separated from depressive-like behavior by evaluating LPS-treated mice in a panel of behavioral paradigms. Our behavioral data indicate that systemic LPS administration caused sickness and mild depressive-like behavior. However, due to the overlapping time course and mild effects on depression-related behavior per se, it was not possible to separate sickness from depressive-like behavior in the present rodent model.

## 1. Introduction

Clinical depression is a devastating, recurrent psychiatric illness that has a lifetime prevalence of 16% [1]. By the year 2030, depression is predicted to become the second leading cause of disability worldwide [2]. Despite its high prevalence and considerable socioeconomic impact, very little is known about the pathophysiology of the disorder. Increasing numbers of studies support the idea that depression is a multifactorial disease with both genetic and environmental factors contributing to disease development [3]. Inflammatory processes may also play a role in the etiology of depression, at least in a subset of susceptible individuals. It has been reported that depressed patients commonly display alterations in their immune system, including impaired cellular immunity and increased levels of proinflammatory cytokines; for reviews, see Schiepers et al. 2005 [4], Dowlati et al. 2010 [5], Blume et al. 2011 [6], and Howren et al. 2009 [7]. Furthermore, depression frequently occurs as a comorbidity of conditions that are characterized by a sustained, systemic inflammation such as rheumatoid arthritis [8, 9], coronary heart disease [10, 11], stroke [12], type 2 diabetes [13], and obesity [14]. Another indication that inflammation and depression are linked comes from clinical observations in which therapeutic administration of the proinflammatory cytokines interleukin-2 and interferon-*α* to cancer or hepatitis C patients resulted in depression in up to half of these patients [15–17].

Bacterial lipopolysaccharide (LPS) is a potent activator of the immune system. Numerous reports have shown that systemic administration of LPS in animals leads to sickness, a behavioral state characterized by symptoms including lethargy, decreased locomotor activity and appetite, anhedonia (the inability to experience pleasure from naturally rewarding activities), sleep disturbances, and increased sensitivity to pain [18, 19]. Several of these symptoms are thought to be very similar to clinically relevant symptoms of depression in humans [20, 21]. Therefore, systemic administration of LPS is frequently used to study inflammation-associated depression in rodents. Some rodent studies report that, 24 h after systemic LPS injection, depressive-like behavior is present without the confounding effects of sickness [22–24]. However, these findings are not consistent across the literature, with some studies describing depressive-like behavior at earlier time points [25, 26] and others still reporting signs of sickness at 24 h [27–30]. Moreover, studies focusing on LPS-induced depressive-like behavior often vary in LPS dose, LPS serotype, application route, and assays used, which makes it difficult to compare results between research groups. In addition, many of these studies only use a single dose of LPS and/or a few time points, thus making it impossible to assess time- and dose-dependent changes in neuroinflammation and behavior. 

The present study aimed at evaluating central effects of peripheral immune activation by combining multiple techniques to quantify neuroinflammation and behavioral changes at several time points after systemic LPS administration. First, transgenic GFAP-luc mice were used to assess the kinetics of LPS-induced astrocyte activation, as marker of neuroinflammation. After confirming the presence of neuroinflammation by immunohistochemistry using the microglial marker ionized calcium-binding adapter molecule 1 (IBA1), serum and brain levels of immune mediators were measured at time points corresponding to glial activation. Finally, LPS-treated mice were tested in a panel of behavioral paradigms to evaluate whether depressive-like behavior could be separated over time from sickness.

## 2. Material and Methods

### 2.1. Animals and LPS

Male NMRI mice were obtained from Charles River Laboratories (France), male wild-type FVB mice from Janvier (France), and GFAP-luc transgenic mice (FVB/N-Tg(Gfap-luc)-Xen) were purchased from Taconic Laboratories (USA). These latter animals express the firefly luciferase gene under the control of a 12 kb murine glial fibrillary acidic protein (GFAP) promoter [31] and are commonly used to noninvasively measure astrocyte activation in the same animal over time [31–36]. Unless mentioned otherwise, animals were housed in groups of 4 in individually ventilated cages (IVC; L × W × H: 36 × 20 × 13 cm; Tecniplast, Italy) under a normal 12:12 h light-dark cycle (lights on at 06:00 a.m. with a 30 min dim and rise phase). Procedure rooms were maintained at a temperature of 22 ± 2°C and a humidity of 54 ± 2%. Food and water were available *ad libitum*. All experimental protocols were approved by the Institutional Ethical Committee on Animal Experimentation, in compliance with Belgian law (Royal Decree on the protection of laboratory animals dd. April 6, 2010) and conducted in facilities accredited by the Association for the Assessment and Accreditation of Laboratory Animal Care (AAALAC).

Lipopolysaccharide (LPS) from *Escherichia coli* (serotype 055:B5) was purchased from Sigma-Aldrich and freshly dissolved in sterile saline prior to injection. 

### 2.2. Bioluminescence

Astrocyte activation in 10-week-old male GFAP-luc mice was monitored before (baseline) and at specific time points (2 h, 6 h, 24 h, 48 h, 72 h, and 96 h) after intraperitoneal (i.p.) administration of either 0, 0.16, or 0.63 mg/kg LPS. Results from a pilot experiment showed that a dose of 2.5 mg/kg LPS in combination with the experimental procedure to measure bioluminescence was lethal in GFAP-luc mice. Therefore, it was decided to use 0.63 mg/kg LPS as the highest dose in this experiment.

To detect the bioluminescent signal, mice were anesthetized by inhalation of 2% isoflurane, shaved on the head, and injected with 126 mg/kg D-luciferin (Promega, product ID E1601) in the tail vein. Three minutes later, the animals were scanned with a charge-coupled device (CCD) camera (IVIS Imaging System 200 Series, Xenogen) mounted on a dark box. The imaging signal was measured in physical units of surface radiance (photons/s/cm^2^/steradian [sr]) using Living Image 3.2 software (Xenogen). Photon emission from the brain was counted using a region of interest (ROI) that was kept constant within the experiment. Bioluminescence coming from the ear was considered to be basal GFAP activity and was excluded from the ROI.

### 2.3. Immunohistochemistry

10-week-old male FVB mice were injected i.p. with vehicle or 0.63 mg/kg LPS, and tissue was collected for immunohistochemical staining 24 h later. Mice were anesthetized with 60 mg/kg sodium pentobarbital (Nembutal) and transcardially perfused with 25 mL heparinised 0.9% saline followed by 50 mL 4% paraformaldehyde (PFA) in 1x phosphate-buffered saline (PBS) (pH 7.4, 4°C). Brains were dissected and postfixed in 4% PFA overnight at 4°C, before being washed twice in PBS and stored in PBS/0.1% NaN3 at 4°C. Free-floating coronal brain sections of 100 *μ*m thickness were cut at the level of the hippocampus (Interaural 1.50 mm, Bregma −2.30 mm, Paxino & Watson, 2001) using a Leica VT1000S vibratome (Leica Microsystems) and were subsequently stored in PBS/0.1% NaN3 at 4°C until use.

For the immunofluorescent staining of IBA1 protein, sections were washed 3 × 5 min in PBS before being incubated in blocking buffer (5% goat serum, 0.3% Triton X 100, and 0.1% bovine specific albumin (BSA) in PBS) for 3 h. Subsequently, sections were incubated overnight at 4°C with a rabbit polyclonal anti-IBA1 (1 : 500, Wako) primary antibody in blocking buffer. The following day, sections were washed 3 × 5 min in PBS before being incubated in PBS-BSA containing the secondary fluorescent antibodies Alexa 555 goat anti-rabbit (1 : 500, Invitrogen), for 2 h at room temperature in the dark. After 3 × 5 min washes in PBS, sections were mounted onto glass slides using a glycerol-based mounting medium containing DABCO (100 mg/mL) and stored in the dark.

A confocal scanning Zeiss Axiovert 100 M microscope was used to obtain fluorescent images. Single images were captured using a Zeiss Plan-Neofluar 10x (NA 0.30) lens. For each animal, two brain sections were analysed, and fluorescent images containing immunopositive cells at the level of the hippocampal dentate gyrus were captured from the 555 nm wavelength. Image analysis software from Zeiss (LSM 510) was used in order to detect changes in the quantity of IBA1 staining levels. Thresholding was used to distinguish positive cells from background. A boundary was drawn around the dentate gyrus of the hippocampus to exclude other regions from quantification. The output of the analysis was “number of pixels.”

### 2.4. Cytokine Measurements

Based on the course of neuroinflammation seen in GFAP-luc mice, it was decided to measure cytokine levels in serum and brain at 2 h, 6 h, and 24 h after LPS administration. For this purpose, 10-week-old male NMRI mice were injected i.p. with 0, 0.63, or 2.5 mg/kg LPS. To reduce animal usage, the 0.16 mg/kg LPS dose was left out as this dose only caused mild GFAP upregulation in the GFAP-luc mice.

At the relevant time points, mice were killed by decapitation, and serum and brain samples were collected. Serum samples were obtained by collecting truncal blood in Vacutainer SST II Advance blood tubes (BD Biosciences, product ID 367955). After being kept for 30 minutes at room temperature, the blood samples were centrifuged (1300 g, 10 min, room temperature), aliquoted, flash-frozen in liquid nitrogen, and stored at −80°C until further use. Within two minutes after decapitation, the brain was isolated from the skull and the hemispheres were separated. They were then weighed, transferred to Tallprep Matrix D tubes (MP Biomedicals, product ID 116973025), flash-frozen in liquid nitrogen, and stored at −80°C until further processing.

A slightly modified protocol adapted from Erickson et al. 2011 [37] was used to extract total protein from brain samples. Briefly, frozen hemispheres were immersed in a 5x volume of extraction buffer (20 mM Tris, 150 mM NaCl, 2 mM EDTA, and 1 mM EGTA) containing a protease inhibitor (Roche, product ID 11873580001) and phosphatase inhibitor cocktail (Roche, product ID 4906837001), and the tissue was homogenized by shaking with a bench top homogenizer (FastPrep-24, MP Biochemicals) for 25 sec. The homogenate was then centrifuged (1000 g, 10 min, 4°C) and supernatant was removed to be centrifuged a second time (20000 g, 40 min, 4°C). Finally, the protein content of each sample was determined using a bicinchoninic acid assay (Sigma-Aldrich), with bovine serum albumin (Sigma-Aldrich, product ID A4503) as a standard. 

Concentrations of interferon-*γ* (IFN-*γ*), interleukin- (IL-) 1*β*, IL-6, IL-10, monocyte chemoattractant protein-1 (MCP-1), and tumor necrosis factor-*α* (TNF-*α*) were simultaneously determined in serum and brain samples using a mouse cytokine/chemokine magnetic bead panel kit from Merck Millipore. This assay is based on Luminex technology in which magnetic beads with a distinct emitting fluorescence pattern are coated onto capture antibodies specific for individual cytokines. All steps in the assay were conducted according to the manufacturer's instructions. A Bio-Plex 200 System (Bio-Rad) was used to measure the fluorescent signal, and the data was analyzed using Bio-Plex Manager 5.0 software (Bio-Rad) with five-parameter logistic regression curve fitting. Cytokine and chemokine concentrations in brain samples were then normalized to the total protein concentration determined for each sample. Cytokines levels below detection limit were assigned a value equal to the lowest detectable value of that cytokine. Cytokine values outside of the average ±3 times standard deviation range were considered outliers and were excluded from all calculations. This happened for less than 2% of all measured cytokines.

### 2.5. Behavioral Tests

Behavioral tests were conducted on 10-week-old male NMRI mice. The open field test (OFT), tail suspension test (TST), and forced swim test (FST) setups were custom-made in-house. In all of these paradigms, groups of naïve mice (*n* = 10/group) were injected i.p. with 0, 0.31, 0.63, or 1.25 mg/kg LPS and tested at either 2 h, 6 h, or 24 h after LPS administration. This dose range of LPS was selected based on results from our previous experiments. The lowest dose of LPS (0.31 mg/kg) was chosen because 0.16 mg/kg LPS only resulted in a mild increase of bioluminescence in the GFAP-luc mice, and it was speculated that a stronger signal was needed to induce behavioral effects. The highest dose of LPS was set to 1.25 mg/kg because 2.5 mg/kg LPS was lethal in the GFAP-luc mice.

The OFT setup consisted of 4 individual arenas (L × W × H: 40 × 40 × 40 cm). Each arena was lit from the top by a lamp producing a light intensity of 800 lux at the bottom. The four arenas allowed testing of four mice at once, while they were separated by nontransparent walls. A video camera with an infrared filter was fixed into the ceiling of each arena, in a way that it covered the entire surface area of that arena. Infrared illumination was provided below the floor of the arenas so mice could be detected and tracked under optimal conditions. Exactly 2 sec after the detection of each individual mouse, tracking of movement was started and performed for 10 min using Noldus EthoVision, version 6.1 (Noldus Information Technology, The Netherlands), with software set up to detect immobility time and distance moved. In this test, exploration behavior of the animal was used to measure locomotor activity.

After single-housing the animals for one day prior to testing, the stress-induced hyperthermia (SIH) paradigm started by measuring the baseline temperature (Temp1). This was done by dipping a rectal probe (Model N9001, Comark Limited, UK) into peanut oil and inserting the probe for 2 cm into the rectum of the mouse while holding the animal in a head-upward position. 15 min later, this procedure was repeated (Temp2) to determine the impact of the mild stress procedure of handling and probe insertion on rectal temperature. In both cases, the rectal probe was kept in place for 15–20 sec in order to standardize stress exposure and reach a stable temperature readout. The mild stress of handling and probe insertion causes a hyperthermic response, and the difference in temperature before and after stress (*dT* = Temp2 − Temp1) reflects the SIH response. This SIH response is suppressed by anxiolytic drugs and is evaluated as a measure of anxiety [38].

The TST consisted of six individual chambers (2 rows with 3 columns; each chamber L × W × H: 14 × 14 × 19.5 cm). A 2.5 cm long hook was fixed to the ceiling of each chamber. The paradigm started by wrapping a piece of tape around the distal part of the tail of each mouse (about 2 cm from the tip) and positioning the mouse upside down when the tape is placed over the hook. The six chambers allowed testing of six mice at once, while they were visually separated by nontransparent walls. A video camera was fixed onto a frame in front of the chambers such that it covered the entire surface of the units. Detection contrast was optimized by using black panels behind the white mice. Exactly 2 sec after detection of each mouse separately, the tracking of movement was started and performed for 6 min. Animals were tracked using Noldus EthoVision, version 6.1, with the software set up to detect immobility time and distance moved (based on center point of gravity of the detected surface). In this test, the animal's immobility was evaluated as a measure of “behavioral despair.”

The FST setup consisted of four independent cylinders (diameter 11 cm) which were automatically flushed and filled with water (10 cm deep, 24-25 degrees Celsius). The four cylinders allowed testing of four mice at once, while they were separated by nontransparent walls. A video camera with an infrared filter was fixed onto a frame in front of the cylinders such that it covered the entire surface area of all four units. Behind the cylinders, infrared illumination was provided to allow optimal detection and tracking of the mice. Exactly 2 sec after detection of each individual mouse, the tracking of movement was started and performed for 6 min using Noldus EthoVision 6.1 software. Immobility time and distance moved (based on center point of gravity of the detected surface) were detected, and the animal's immobility was evaluated as a measure of “behavioral despair.”

In the sucrose preference test (SPT), animals were single-housed in special Plexiglas IVC (L × W × H: 35 × 31 × 16 cm; Tecniplast, Italy) fitted with two 250 mL plexiglass drinking bottles (Tecniplast). Each bottle contained either filtered tap water or a sucrose solution (1, 2, 5, or 10%). The experiment consisted of a *familiarization* and a *test phase*. During the *familiarization phase,* all animals were presented for 24 h with two water-filled bottles (W/W) on day (D) 1 and D3, or one water- and one sucrose-filled bottle (W/S) on D2 and D4. The bottles were removed between 08:00 and 09:00 a.m. each day and weighed using Software Wedge for Windows 1.2 (TAL Technologies). Animals were weighed, and freshly prepared bottles were put into the cages. The amount drunk by a mouse was determined by subtracting the weight of the bottle at the start of the observation period and at the end 24 h later (taking fluid density as 1 g/mL). Total fluid intake was taken as the total change in volume from both bottles combined, while the preference for sucrose was calculated as a percentage of consumed sucrose solution of the total fluid intake. A total fluid intake that was greater than the mean +2x standard deviation was considered to be an invalid measure that probably resulted from leaking bottles. Invalid measures were replaced by the mean of all the bottles either on the relevant side (for W/W) or for either water or sucrose (for W/S). This happened in less than 1% of all bottle measurements. The *test phase* of the experiment started 3 days after the *familiarization phase* by injecting the mice with either vehicle or 0.63 mg/kg i.p. LPS. This dose of LPS was chosen because it had a clear effect on neuroinflammation and sickness behavior in the previous experiments. Immediately after LPS administration, the mice were presented with W/S for 24 h. This procedure was repeated for 3 consecutive days. Total intake volume was evaluated as a primary measure for sickness behavior (reduction versus normal daily intake), while sucrose preference was used as a measure for anhedonia.

### 2.6. Statistical Analysis

Data were analyzed using SPSS Statistics software (Version 20 for Windows, IBM Inc.). Analysis of variance (ANOVA) or, when appropriate, ANOVA with repeated measure analysis (rmANOVA) was performed. A Greenhouse-Geisser correction epsilon (*ε*) was used for repeated measures analysis to correct for potential violation of the sphericity assumption [39]. This correction multiplies both the numerator and the denominator degrees of freedom by epsilon, and the significance of the *F*-ratio is evaluated with the new degrees of freedom, resulting in a more conservative statistical test. When significant, post hoc comparisons were made by using an independent samples *t*-test with a Bonferroni-corrected *P* value. Significance was accepted for the ANOVAs and post hoc comparisons when *P* < 0.05. All data are expressed as mean ± standard error of the mean (SEM).

Bioluminescent signals in the GFAP-luc mouse were analyzed by rmANOVA using dose (3 levels: 0, 0.16, and 0.63 mg/kg LPS) as a between-subjects factor and time (7 levels: BL, 2 h, 6 h, 24 h, 48 h, 72 h, and 96 h) as a within-subject factor. Number of pixels in IBA1 positive cells were analyzed by ANOVA using dose (2 levels: 0 and 0.63 mg/kg LPS) as between-subjects factor. Cytokine levels were analyzed by separate ANOVAs for each cytokine with dose (3 levels: 0, 0.63, and 2.5 mg/kg LPS) and time (2 h, 6 h, and 24 h) as between-subjects factor. Distance moved in OFT and immobility time in TST and FST were analyzed using separate ANOVAs with dose (4 levels: 0, 0.31, 0.63, and 1.25 mg/kg LPS) as between-subjects factor. For the SIH procedure, both temperatures (Temp1 and Temp2) were analyzed as a repeated measure and dose (4 levels: 0, 0.31, 0.63, and 1.25 mg/kg LPS) as a between-subjects factor. Total volume intake and sucrose preference in both phases of the SPT were separately analyzed using rmANOVA. In the *familiarization phase*, flavor (2 levels: W/W and W/S) and repeat (2 levels: first test and retest) were used as within-subject factor and treatment group (5 levels: 1%, 2%, 5%, and 10% sucrose/LPS and 10% sucrose/vehicle) as a between-subjects factor. For the *test phase*, treatment group (5 levels: 1%, 2%, 5%, and 10% sucrose/LPS and 10% sucrose/vehicle) was again used as a between-subjects factor and time (3 levels for total volume intake and sucrose preference: D8, D9, and D10) as a within-subject factor.

## 3. Results

### 3.1. Effect of Systemic LPS Administration on Brain Bioluminescence in GFAP-luc Mice

**Figure 1:**
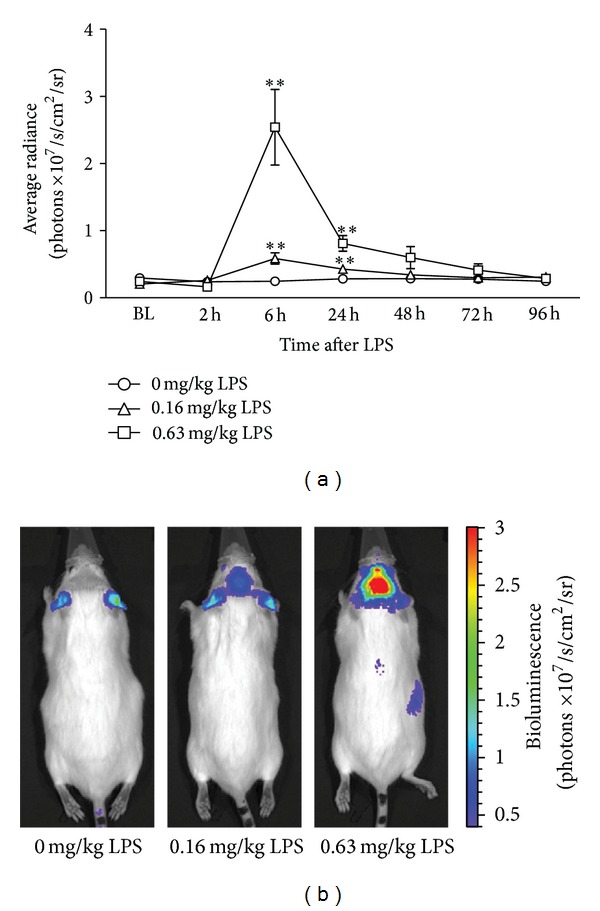
Intraperitoneal administration of LPS caused a dose- and time-dependent increase in brain bioluminescent signal in GFAP-luc transgenic mice (a). A clear LPS-induced bioluminescent signal was visible in the brain, as seen on representative images taken from animals treated with different doses of LPS at 6 hours after injection (b). The color on the image represents the number of photons emitted from the animal per second, as indicated in the color scale on the right. Graphs are plotted as mean + SEM (*n* = 8 per group). Data were analyzed by rmANOVA followed by independent samples *t*-test. **P < 0.01 compared to 0 mg/kg LPS.

Factorial rmANOVA of photons emitted per second in the brain region of interest revealed a significant time × dose interaction (*F*(12, 96) = 15.0, *P* < 0.001, *ε* = 0.18). Post hoc analysis showed that, at 6 h after LPS, a strong and brain-specific bioluminescent signal was present in mice treated with 0.63 mg/kg, while, at this time point, a more moderate but still significant signal was evoked in the 0.16 mg/kg LPS group (Figure 1). For both groups, there was still a significant increase in brain bioluminescence at 24 h, but no longer at 48 h after LPS. Bioluminescence coming from the ears did not change during the experiment and was considered to be a background signal.

**Figure 2:**
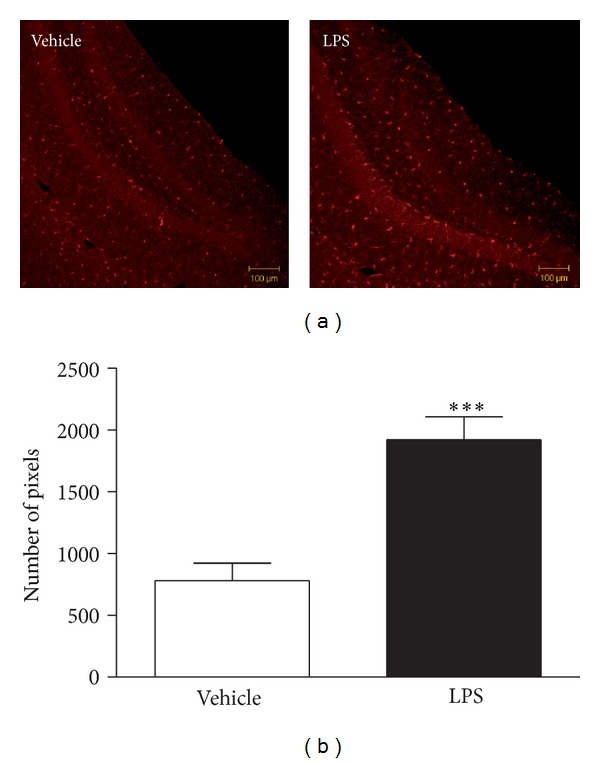
Peripheral LPS injection (0.63 mg/kg, i.p.) increased IBA1 immunoreactivity, a marker of microglial activation, in the hippocampal dentate gyrus at 24 h after administration. Representative images (10x) (a), quantified images of *n* = 10 per group (b). Graph is plotted as mean + SEM. Data were analyzed by ANOVA followed by independent samples *t*-test. ***P < 0.001 compared to vehicle.

Because the bioluminescence data revealed a significant LPS-induced GFAP upregulation, it was decided to confirm the presence of glial activation by immunohistochemistry using a microglial marker. Therefore, the expression of IBA1 was quantified in the hippocampal dentate gyrus at 24 h after systemic administration of vehicle or 0.63 mg/kg LPS. This brain structure was chosen because it is associated with stress and depression [40–42] and commonly studied in models of LPS-induced neuroinflammation [43, 44]. Although astrocyte activation in the GFAP-luc mouse peaked at 6 h after LPS, it was decided to quantify IBA1 expression at 24 h as some studies reported depressive-like behavior in the absence of sickness at this time point [23, 24]. Furthermore, astrocyte activation was still increased in LPS-treated mice at 24 h, thereby indicating the relevance of measuring glial activation at this point. Factorial ANOVA indicated a significant effect of dose (*F*(1,18) = 23.9, *P* < 0.001), and post hoc analysis showed that the pixel number of IBA1 positive cells was significantly higher in mice that received LPS when compared to vehicle-treated mice (Figure 2).

### 3.2. Effect of Systemic LPS Administration on Serum and Brain Cytokine Levels

**Figure 3:**
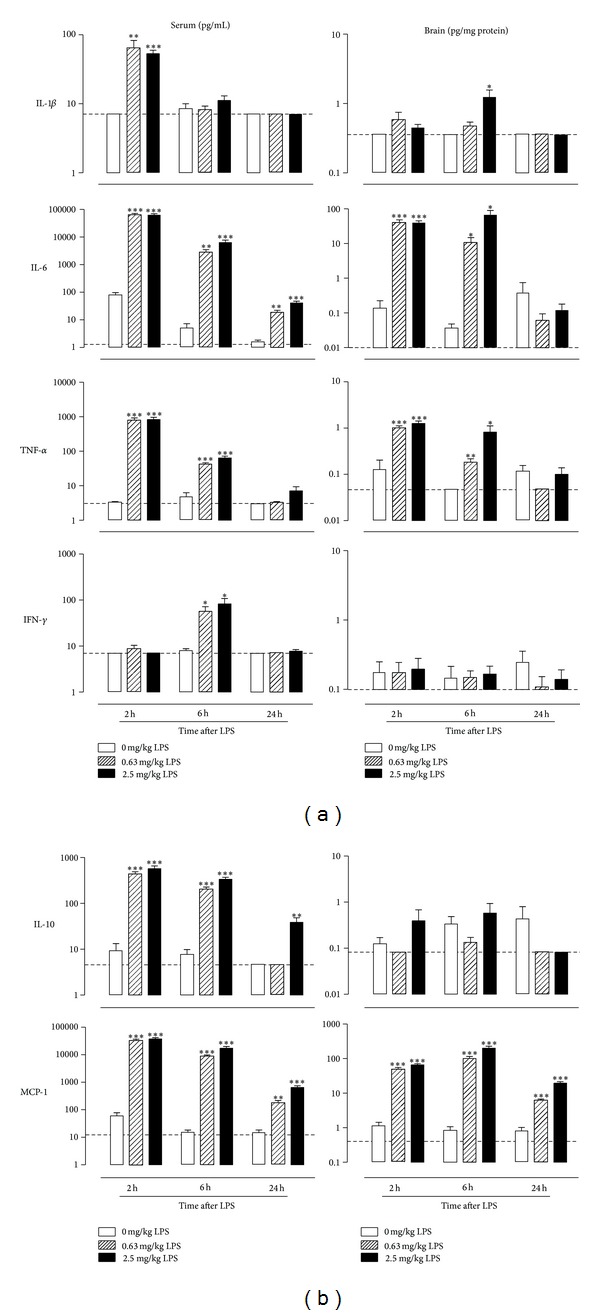
Peripheral LPS administration transiently increased cytokine levels in serum and brain. Comparison of selection of cytokines and one chemokine (MCP-1) in serum (left) and brain (right) after i.p. LPS administration. Dashed lines indicate the detection limit of measured cytokine. Note that serum concentrations are expressed in pg/mL, while brain levels are shown in pg/mg protein. Graphs are plotted as mean + SEM (*n* = 12 per group). Data were analyzed by ANOVA followed by independent samples *t*-test. *P < 0.05, **P < 0.01, ***P < 0.001 compared to 0 mg/kg LPS.

For all cytokine levels measured in serum, a significant time × dose interaction was found (IL-1*β*: *F*(4,96) = 6.9, *P* < 0.001; IL-6: *F*(4,97) = 40.9, *P* < 0.001; TNF-*α*: *F*(4,95) = 18.8, *P* < 0.001; IFN-*γ*: *F*(4,98) = 4.9, *P* < 0.01; IL-10: *F*(4,95) = 14.3, *P* < 0.001; and MCP-1: *F*(4,95) = 22.7, *P* < 0.001). Post hoc analysis demonstrated that serum cytokine levels in vehicle-treated mice were undetectable or minimal at all time points (Figure 3, left column). Serum levels of IL-1*β*, IL-6, TNF-*α*, IL-10, and MCP-1 increased significantly after administration of 0.63 or 2.5 mg/kg LPS, peaking at 2 h after administration and gradually decreasing over time. Serum IFN-*γ* levels in LPS-treated animals followed a slightly different time course as the peak for this cytokine was reached at 6 h after LPS. At 24 h after LPS administration, the serum levels of IL-1*β*, TNF-*α*, and IFN-*γ* had returned to baseline values, while IL-6 and MCP-1 were still elevated in 0.63 and 2.5 mg/kg LPS-treated animals and IL-10 only in 2.5 mg/kg LPS-treated mice.

A significant time × dose interaction was found on brain levels of IL-1*β*, IL-6, TNF-*α*, and MCP-1 (IL-*β*: *F*(4,98) = 5.6  *P* < 0.05; IL-6: *F*(4,96) = 9.7, *P* < 0.001; TNF-*α*: *F*(4,97) = 8.2, *P* < 0.001; and MCP-1: *F*(4,97) = 24.3, *P* < 0.001), but no significant effect of time or dose could be detected on IFN-*γ* or IL-10 brain levels. Comparable to the time course of their release in serum, brain levels of IL-6, TNF-*α*, and MCP-1 peaked at 2 h posttreatment in mice exposed to 0.63 and 2.5 mg/kg LPS (Figure 3, right column). Apart from MCP-1 levels, which were still elevated in the brains of LPS-treated mice at 24 h, all brain cytokine levels had returned to baseline values at 24 h after LPS injection. IL-1*β* was slightly, but significantly, increased at 6 h in the brains of mice that received 2.5 mg/kg LPS, but not at 0.63 mg/kg.

### 3.3. Effect of Systemic LPS Administration on Behavior across a Panel of Sickness, Anxiety, and Depressive-Like Behavior Assays

**Figure 4:**
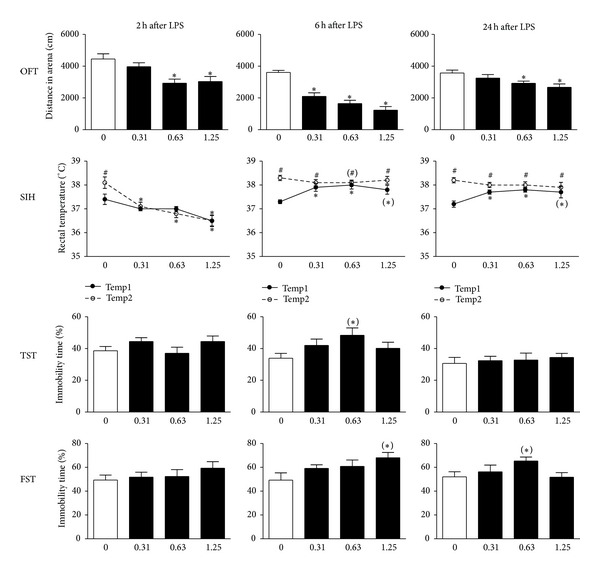
Intraperitoneal injection of LPS caused sickness, but no clear depressive-like behavior is observed. Peripheral immune activation caused a dose- and time-dependent reduction in locomotor activity in the open field test (OFT). However, a single i.p. injection of LPS did not induce clear anxiety or depressive-like behavior in the stress-induced hyperthermia (SIH) test, tail suspension test (TST), or forced swim test (FST). Graphs are plotted as mean + SEM (*n* = 10 per group). Data were analyzed by multivariate ANOVA followed by independent samples *t*-test. *P < 0.05 compared to 0 mg/kg LPS group, ^(∗)^0.05 > *P* > 0.1 compared to 0 mg/kg LPS group, ^#^ *P* < 0.05 compared to Temp1 (SIH) ^(#)^0.05 > *P* > 0.1 compared to Temp1 (SIH).

The total distance travelled in the open field test is a general measure for exploration and can be used as a marker of sickness behavior. Factorial ANOVA revealed a significant main effect for the factor dose at all time points tested (2 h: *F*(3,36) = 6.6, *P* < 0.01; 6 h: *F*(3,35) = 23.7, *P* < 0.001; and 24 h: *F*(3,36) = 4.3, *P* < 0.05). Post hoc analysis demonstrated that animals exposed to 0.63 or 1.25 mg/kg LPS showed reduced locomotor activity at 2 h posttreatment (Figure 4, OFT). At 6 h, all doses of LPS led to a reduced distance travelled in the OFT, while at 24 h only mice treated with 0.63 or 1.25 mg/kg LPS showed a significant reduction in exploration when compared to vehicle-treated mice.

The stressed-induced hyperthermia paradigm reflects a physiological response to mild stress exposure and is sensitive to treatment with anxiolytic drugs [38]. The measure for anxiety in this paradigm is the increase in body temperature in response to the mild stress of measuring rectal temperature. rmANOVA revealed a significant interaction for stress × dose at all time points tested (2 h: *F*(3,36) = 5.4, *P* < 0.01; 6 h: *F*(3,36) = 14.0, *P* < 0.001; and 24 h: *F*(3,36) = 21.3, *P* < 0.001). Post hoc analysis demonstrated that, at 2 h after LPS, there was a dose-dependent decrease in both Temp1 and Temp2, which was significant for Temp1 at 1.25 mg/kg and for Temp2 in all LPS-treated mice (0.31, 0.63, and 1.25 mg/kg LPS) (Figure 4, SIH). As LPS lowered both Temp1 and Temp2, there was no SIH effect in any of the LPS-treated mice, while it remained significant in control animals. At 6 h and 24 h following LPS, Temp1 was significantly increased in LPS-treated mice (0.31, 0.63, and 1.25 mg/kg), but there was no significant difference in Temp2 between LPS-challenged and control mice. At these time points, there was a significant SIH effect in all groups.

In the tail suspension test, behavioral despair was evaluated by measuring the time during which an animal remains immobile after being suspended by the tail. Factorial ANOVA revealed a trend for the factor dose at 6 h after LPS (*F*(3,35) = 2.3, *P* = 0.09), but no statistical significance was found at 2 h or 24 h. Explorative post hoc analysis revealed that, at 6 h after LPS administration, mice treated with 0.63 mg/kg LPS, but not 0.31 or 1.25 mg/kg LPS-treated animals, showed a slightly increased immobility time (Figure 4, TST). 

Behavioral despair in the forced swim paradigm was evaluated by measuring the time during which a rodent remains immobile after being placed in a water-filled cylinder from which it cannot escape. At 6 h after LPS, a trend was found for the factor dose (*F*(3,35) = 2.6, *P* = 0.07), but no statistical significance was found for any of the other time points. Explorative post hoc analysis revealed that, at 6 h after administration, LPS induced a slight increase in immobility time that was significant in the 1.25, but not in the 0.31 or 0.63 mg/kg LPS-treated animals (Figure 4, FST). At 24 h after LPS injection, animals treated with 0.63 mg/kg remained immobile for a longer period than control animals. However, this increased immobility at 24 h after injection was not seen in mice exposed to 0.31 or 1.25 mg/kg LPS.

**Figure 5:**
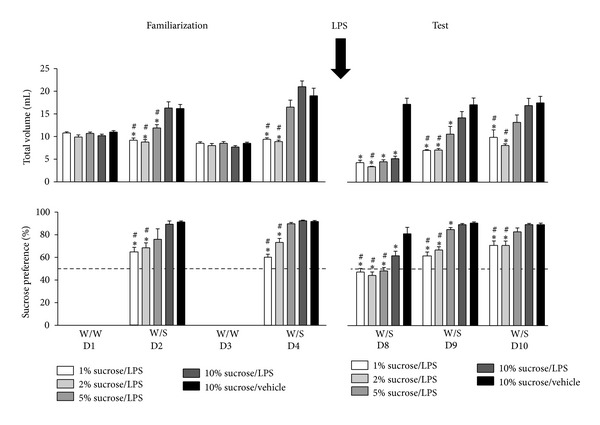
Intraperitoneal injection of LPS caused a transient reduction in total volume intake and sucrose preference in the sucrose preference test. During the familiarization phase of the experiment (left), animals were familiarized to the experimental setup. On day (D) 1 and D3, mice were exposed to 2 bottles of water (W/W), while on D2 and D4 one bottle contained water and the other bottle was filled with a 1, 2, 5, or 10% sucrose solution (W/S). Voluntary consumption of water or sucrose was measured during a period of 24 h for up to 3 days after systemic LPS administration (D8–D10). Dashed lines indicate chance level for sucrose preference. Graphs are plotted as mean + SEM (*n* = 10 per group). Data were analyzed by repeated measures ANOVA followed by independent samples *t*-test. *P < 0.05 compared to 10% sucrose/vehicle group, ^#^ *P* < 0.05 compared to 10% sucrose/LPS group. W/W: water/water; W/S: water/sucrose.

The sucrose preference test, in which the preference of an animal for a sweetened solution versus water is measured, is a commonly used rodent model to evaluate anhedonia. Our experiment consisted of two phases. The purpose of the *familiarization phase* was to assess normal daily intake volume, familiarize the animals with exposure to sucrose, and determine the effect of different sucrose concentrations on sucrose preference. rmANOVA revealed that, for total intake volume during the *familiarization phase,* there was a flavor × repeat × group interaction (*F*(4,45) = 5.8, *P* < 0.001). Furthermore, a main effect of group (*F*(4,45) = 20.6, *P* < 0.001) was found for sucrose preference. Post hoc analysis demonstrated that total intake volume in the *familiarization phase* increased significantly when animals were exposed to both sucrose and water (D2 and D4), but only when the animals were retested (D4) with a sucrose concentration of 5 or 10% (Figure 5, top left panel). The levels of sucrose preference correspond to these findings, as sucrose preference was significantly lower in mice exposed to 1% or 2% sucrose, but not in mice receiving 5% sucrose, when compared to mice exposed to 10% sucrose (Figure 5, bottom left panel).

In the *test phase*, the effect of i.p. LPS administration on total intake volume and sucrose preference was assessed over time. rmANOVA revealed that there was a strong time × group interaction for total volume intake (*F*(8,90) = 8.5, *P* < 0.001, *ε* = 0.86). Post hoc analysis indicated that, in the first 24 h after administration (D8), LPS reduced the total intake volume to less than half of the normal daily water intake, suggesting suppression of drinking as a consequence of sickness (Figure 5, top right panel). On the second day after LPS administration (D9), the LPS-induced reduction in total volume intake was no longer present in mice exposed to 10% sucrose solution, while it remained in the mice receiving 1, 2, or 5% sucrose. At D10, the total intake volume of all mice had returned to baseline levels.

For sucrose preference in the *test phase*, rmANOVA revealed a time × group interaction (*F*(8,90) = 4.3, *P* < 0.001, *ε* = 0.84). In line with the total intake volume data, post hoc analysis demonstrated that, on D8, the sucrose preference was reduced in all LPS-treated animals (Figure 5, bottom right panel). In the following days, sucrose preference recovered depending on the sucrose concentration; as on D9, the sucrose preference for LPS-treated mice receiving 10% sucrose had returned to pre-LPS values, while for mice receiving 1, 2, or 5% sucrose this took up to D10.

## 4. Discussion

Based on the complexity and heterogeneity of depression, it is likely that several interacting systems underlie its pathogenesis. Findings from clinical studies indicate that inflammatory processes are associated with depression, at least in certain clinical subpopulations. For example, subsets of depressed patients show alterations of their peripheral immune system [4–7], and depression often occurs as a comorbidity in patients suffering from conditions characterized by a sustained, systemic inflammation [8–14]. Moreover, therapeutic stimulation of the immune system leads to depression in up to half of cancer and hepatitis C patients receiving proinflammatory cytokine treatment [15, 17].

Inflammation-associated depression is often studied in rodents by systemic administration of bacterial LPS, which is a potent activator of the immune system. Results from previous rodent studies indicate that systemic application of a single bolus of LPS leads to sickness behavior that peaks at 2–6 h, gradually fades over time, and is attenuated at 24–48 h after LPS injection (for a review, see Dantzer et al. 2008 [20]). There are some indications that depressive-like behavior can be separated from sickness 24 h after systemic LPS administration [22–24]. Contrastingly, other studies showed that LPS-induced signs of sickness are still present at that time [27–30], making it difficult to compare results from different labs. Other factors complicating the interpretation of the existing literature include the difference in experimental design between studies and the use of only a single dose of LPS and/or a few time points. Consequently, assessing time- and dose-dependent changes in neuroinflammation and behavior following systemic LPS administration is not straightforward.

The present study was therefore designed to evaluate central effects of systemic LPS administration at several time points by combining multiple techniques to quantify neuroinflammation and behavioral changes. To our knowledge, such an extended and multidisciplinary approach has not yet been reported in this field. First, the kinetics of neuroinflammation following peripheral immune activation were assessed using a transgenic mouse line that carries the luciferase gene under the transcriptional control of the mouse GFAP promoter. GFAP is an intermediate filament protein that is predominantly expressed by astrocytes and is upregulated when astrocytes are activated [45]. This makes the bioluminescent GFAP-luc mouse model an ideal tool to quantify astrocyte activation, as marker of neuroinflammation, in living mice. Systemic LPS administration to these GFAP-luc mice led to a time- and dose-dependent increase in brain bioluminescence that peaked at 6 h after LPS administration and then gradually faded over time. The upregulation of GFAP at 6 h after systemic LPS injection demonstrates that astrocytes respond rapidly to a peripheral immune challenge. This early response of brain cells to peripheral immune activation has also been shown in another bioluminescent mouse model where systemic LPS administration induced a time- and dose-dependent increase in the expression of CCAAT enhancer binding protein (C/EBP), a regulator of inflammation [46]. However, C/EBP upregulation peaked at 24 h after LPS, a time point at which GFAP expression has decreased substantially, suggesting that astrocyte activation might be an early and short-lasting response to peripheral immune stimulation, while other inflammatory processes in the brain persist. GFAP-luc mice treated with the highest dose of LPS (2.5 mg/kg) died during scanning at 6 h after LPS. This was unexpected as the same dose was not lethal in NMRI mice tested throughout the rest of the study. One possible explanation for this discrepancy may be a strain-related difference in LPS sensitivity as previously described in models for acute lung injury [47] and inflammation-induced depression [48]. Other potential causes for the unexpected mortality in GFAP-luc mice treated with a high dose of LPS might be found in the experimental procedure to measure bioluminescence. It is possible, for example, that the toxic effects of isoflurane and/or potassium bound to luciferin become lethal in combination with a high dose of LPS.

To confirm glial activation using a different technique and another glial cell type, IBA1 expression was quantified in the hippocampal dentate gyrus of LPS-treated FVB wild-type mice. This brain structure is associated with stress and depression [40–42] and commonly studied in models of LPS-induced neuroinflammation [43, 44]. IBA1 is expressed in microglia, and its expression is elevated under pathological conditions [44, 49–51]. Consistent with astrocyte activation found in the GFAP-luc mouse, LPS-treated FVB wild-type mice showed a robust increase in IBA1 reactivity in the dentate gyrus. These results indicate that microglia, in addition to astrocytes, also show signs of activation in response to systemic LPS administration and are in line with previous reports of increased IBA1 immunoreactivity in the hippocampus of LPS-treated mice [44, 52].

Acute systemic LPS administration is known to induce a transient release of cytokines in the periphery and CNS [37, 53, 54]. In agreement with the literature, the present study showed that serum levels of cytokines that are involved in the acute phase response of inflammation (IL-1*β*, IL-6, and TNF-*α*) were upregulated 2 h after peripheral LPS administration. Serum levels of IFN-*γ*, however, were only increased 6 h posttreatment, suggesting that the release of this cytokine was probably not triggered by LPS directly, but by downstream effects of earlier released cytokines. Serum levels of most proinflammatory cytokines had returned to baseline values at 24 h. However, at this time point, the serum levels of IL-6 and the chemokine MCP-1 were still slightly elevated in all LPS-treated mice, demonstrating that the immune system was still mildly activated in the periphery. IL-10, an anti-inflammatory cytokine that plays a role in regulating the intensity and duration of the inflammatory response, remained elevated in the serum of mice treated with a high dose of LPS. The fact that IL-10 levels were no longer elevated at 24 h in the serum of mice treated with a low LPS dose indicates that anti-inflammatory pathways return to baseline quicker after a less pronounced immune activation.

Cytokines from the periphery can pass the blood-brain barrier (BBB) and reach the brain through humoral, neural, and cellular pathways [55–57]. LPS has been shown to affect BBB permeability in several ways. Apart from early findings that LPS disrupts the BBB, LPS is now also known to exert direct effects on tight junction regulation [58] and cytokine release from endothelial cells in the brain [59]. However, the present study did not measure the integrity of the BBB and did not account for the fact that cytokines from the periphery can enter the brain through a leaky BBB. Despite this limitation, it was found that the time-dependent brain profiles of IL-6, TNF-*α*, and MCP-1 matched the serum profile, suggesting that these cytokines are expressed at a similar rate in the brain and/or that they can easily cross the BBB. Although IL-1*β* is known to pass the BBB [60], its brain levels were only significantly elevated at 6 h after LPS in mice receiving 2.5 mg/kg, but not in mice receiving 0.63 mg/kg LPS. These findings are in line with results described by Puentener and colleagues [54], who did not find an increase in IL-1*β* brain levels at 3 hours after acute i.p. administration. Erickson and Banks, in contrast, described an elevation of IL-1*β* brain levels in mice at 24 h after a single dose of LPS [37]. The present study was unable to reliably detect brain levels of IFN-*γ* and IL-10. Based on the large number of samples below detection limit in all treatment groups, this was likely due to a sensitivity issue and not to lack of cytokine levels in the brain. However, the strong increase in brain levels of IL-6, TNF-*α*, and MCP-1 confirmed that systemic LPS administration leads to a proinflammatory status in the brain. The brain levels of most cytokines returned to baseline at 24 h, while levels of the chemoattractant MCP-1 remained elevated. This indicates that there is still mild neuroinflammation present at this time point and coincides with the time course of astrocyte activation in the GFAP-luc mouse model and IBA1 immunoreactivity in the dentate gyrus of LPS-treated mice. This study did not account for regional differences of cytokine profiles in the brain. However, results from several other studies have pointed out that there might be a spatiotemporal component to LPS-induced cytokine production in the brain [53, 61–63]. Future research focusing on the identification of local changes in neuroinflammation may help to identify brain areas that are involved in inflammation-associated depression.

In addition to evaluating the LPS-induced peripheral and central immune responses as described previously, the second aim of this study was to investigate the main and side effects of peripheral LPS administration on behavior. Some indications already exist that, at 24 h after acute peripheral LPS injection, depressive-like behavior can be observed in rodents. However, the nature and characteristics of LPS-induced sickness behavior can substantially confound measurements of depressive-like behavior in commonly used paradigms. For example, sick animals show reduced motor activity which can confound measures of immobility, used to estimate despair in inescapable conditions (e.g., tail suspension and forced swim test) [20]. Therefore, studies focusing on depressive-like behavior should also include measures of sickness. Several groups have already used a combination of behavioral tests for that purpose. In some of these studies, a time window was identified in which sickness had dissipated while depressive-like behavior was still present. However, findings from different labs often vary. Some groups showed that LPS-treated mice display increased immobility in the tail suspension and forced swim test at 24 h, a time point at which motor activity in the open field test had returned to baseline [23, 24]. In contrast, other groups still observed reduced locomotor activity as an indication of sickness at this time after LPS administration [29, 30, 64]. Studies measuring sickness by evaluating social behavior are also not clear on the duration of LPS-induced sickness. Some groups, for example, have shown that social behavior returned to normal at 24 h after LPS [22, 29, 65], while others still report deficits in social behavior at this time point [27]. Hence, we evaluated the dose dependency and time course of LPS-induced changes in behavior across a panel of assays that are commonly used to study sickness, anxiety, and depressive-like behavior in rodents. Sickness, as measured by decreased locomotion in the OFT, occurred at 2 h after LPS treatment and had dissipated at 24 h in mice treated with a low dose of LPS. Animals treated with higher doses of LPS, however, still showed reduced locomotor activity at this point, indicating that sickness remained present in these mice. This timing coincided with signs of sickness seen in the SIH test where the baseline temperature (Temp1) of LPS-treated mice remained elevated at 24 h after LPS, thereby confounding measures of anxiety (dT). Depressive-like effects as evaluated by immobility time in the TST and FST were very low at all measured time points and can be considered biologically irrelevant here due to the cooccurrence of sickness. Furthermore, it is worth to note that we used naïve mice at each time point in our behavioral tests to avoid differences in confounding habituation effects (due to repeated testing) between sick and control animals.

From the sucrose preference experiment results, it becomes clear that the concentration of sucrose is a key factor for sucrose preference in mice. As seen on the last day of the *familiarization phase *(D4), the sucrose preference increased with sucrose concentration, with a ceiling effect being reached at 5% sucrose. Mice exposed to 5–10% sucrose also clearly drank much more than their normal daily intake, that is, on a day where they were exposed to water only. However, this was not the case in mice receiving 1-2% sucrose, showing that the total volume intake also depends on the sucrose concentration. Moreover, our data reveal that LPS reduced sucrose preference in a time-dependent manner. These findings are in line with previous results showing that LPS administration to mice decreased their sucrose consumption [23] and sucrose preference [22] for up to 2 days after administration, while leaving their water and food intake unaltered [23]. Despite the fact that, in our study, there was also no difference in water intake between treatment groups during the first 24 h after LPS administration (data not shown), it is important to mention that, at this time, the total volume intake in LPS-treated mice was reduced to approximately half of the normal daily intake. This suppressed drinking suggests that sickness still seems to be a confounding factor when measuring sucrose preference during the first 24 h after LPS administration and points out that caution is needed when interpreting LPS-induced reduction in sucrose preference as a measure of anhedonia.

Our data clearly show that acute systemic administration of LPS leads to a strong but ephemeral activation of the peripheral immune system with accompanying neuroinflammation and behavioral effects. Inflammation-associated depression in humans, however, is linked to chronic, persistent inflammation [21, 66]. This makes acute LPS administration to mice a less attractive translational model for inflammation-associated depression in humans. Interestingly, Kubera and coworkers recently described a mouse model in which repeated LPS injections given at one-month intervals induced a chronic state of anhedonia, indicating that chronic LPS administration might be a more relevant approach to induce depressive-like behavior [67]. In that study, the prolonged anhedonia in response to repeated LPS administration was only observed in female, but not in male mice. In another study, a less elaborate model of repeated LPS administration was shown to induce depressive-like behavior in absence of sickness in male rats [61]. It is possible that, as hypothesized for the human situation, a chronic inflammatory tone is needed to elicit depressive-like behavior in rodents. However, future work is needed to evaluate whether repeated LPS administration in rodents is a more valid model of inflammation-associated depression.

## 5. Conclusion

The present set of experiments using various assays and readouts confirmed that there is a strong crosstalk between the immune system and the brain, both on a neuroimmune and neurobehavioral level. Acute systemic LPS administration in mice caused a marked but transient increase in pro- and anti-inflammatory cytokines in the periphery. The time course of the systemic inflammation coincided with neuroinflammation as seen by astrocyte activation in GFAP-luc mouse, increased IBA1 immunoreactivity in the hippocampus, and elevated cytokine levels in the brain. Moreover, thorough investigation of several primary parameters across a panel of behavioral assays showed that systemic LPS administration induced sickness lasting for up to 48 hours. This time-dependent profile coincided with mild depressive-like behavior. However, due to overlapping time windows and rather mild effects on depressive-like behavior per se, it is not possible to separate sickness from depressive-like behavior in the present rodent model.
